# Exploring the dynamics and trends of carbon emission spatiotemporal patterns in the Chengdu–Chongqing Economic Zone, China, from 2000 to 2020

**DOI:** 10.1038/s41598-024-67204-5

**Published:** 2024-07-16

**Authors:** Lu Che, Sidai Guo, Yangli Li, Yihao Zhu

**Affiliations:** 1 School of Environment and Resources, Southwest University of Science and Technology, Mianyang, 612000 China; 2 School of Economics and Management, Southwest University of Science and Technology, Mianyang, 612000 China; 3 School of Civil Engineering and Architecture, Southwest University of Science and Technology, Mianyang, 612000 China

**Keywords:** Carbon emissions, Spatiotemporal variations, Qualitative analysis, Quantitative analysis, Ecology, Environmental sciences

## Abstract

Analysis of the spatial–temporal pattern and trend of carbon emissions provides an important scientific basis for the development of a low-carbon economy. Based on the corrected NPP-VIIRS and DMSP/OLS nighttime light data, a carbon emission model for the Chengdu–Chongqing Economic Zone (CCEZ) in China is constructed. Furthermore, the article establishes an integrated qualitative and quantitative research system. The qualitative results show that at the city and county scales, the high carbon emission areas and counties are mainly distributed in Chengdu and Chongqing, while the low carbon emission areas are concentrated in the marginal cities of the CCEZ and the counties with low levels of industrialization around the Sichuan Basin. The high-carbon emission zone tended to expand to the north, and the low-carbon emission zone tended to expand to the south. At the grid scale, the carbon emissions of the CCEZ fluctuated and increased from 2000 to 2020, forming a trend connected with those of the central city, with high carbon emissions at the core and radiating outward expansion. Quantitative analysis revealed that carbon emissions at the county and grid scales exhibited a significant positive global spatial correlation, and the overall correlation degree exhibited an increasing trend.

## Introduction

China ranks first in global CO_2_ emissions and accounted for 27% of global CO_2_ emissions in 2017^1^. China is committed to increasing its national contributions and aims to peak its carbon dioxide emissions by 2030 and achieve carbon neutrality by 2060, guided by policies to promote green and low-carbon development nationwide in China^2,3^. Therefore, it is crucial to accurately measure and analyze the spatial and temporal changes in carbon emissions in different regions and to reveal the underlying mechanisms to achieve energy conservation, emission reduction, and scientific planning for green and low-carbon goals^4^.

In recent years, research on “carbon emissions” in the field of environmental and economic geography in China has mainly relied on data released by government departments. It is relatively difficult to study areas with lagging, missing or microscopic data. Different regions often face different carbon emission issues due to differences in resource endowments, population, and energy structure^5,6^. Therefore, how to use new data sources for research should be the focus of current research on carbon emissions in the field of geography^7,8^. The data obtained from the US Defense Meteorological Satellite Program (DMSP) have a long time span and wide coverage and can capture the weak light intensity on the Earth’s surface. It is an important data source for obtaining urban information^9,10^. Continuous high-resolution nighttime light data can clearly detect human activities. Many scholars have conducted estimation studies on electricity consumption^11,12^ and carbon emissions based on these data^13–15^. Some scholars first constructed a model of nighttime light data and electricity consumption and confirmed their correlation. They discussed the advantages of nighttime light mapping for carbon dioxide emissions and further analyzed the dynamic changes in carbon emissions within large cities using nighttime light data^16–18^. Elvidge^19^ first confirmed that nighttime light data can be used to model the spatial distribution of carbon dioxide. Doll^20^ obtained the first global carbon dioxide grid with a resolution of 1° × 1° based on nighttime light data. Wang, YP^21^ analyzed the spatial–temporal pattern changes in China’s carbon emissions at multiple scales based on DMSP/OLS data. Wang, GJ^22^ used DMSP-OLS and NPP-VIIRS data to establish a fitting equation between urban-scale light and carbon emissions in China, which corresponded to the carbon emissions of each subregion, objectively demonstrating the geographical distribution of carbon emissions. Li^23^ analyzed the spatial–temporal pattern and determining factors of energy-related carbon emissions in the Yellow River Basin using remote sensing data.

Overall, research on carbon emissions has focused mainly on the national, provincial, and municipal levels. In addition, the research methods mainly include the spatial autocorrelation model, stochastic block model, and spatial Markov chain model. Some scholars have further explored the factors influencing carbon emissions^24–26^. Most of the above studies mainly used DMSP/OLS or NPP-VIIRS data, and few studies integrated nighttime light data from these two different time sequences. Moreover, most of the studies are large in scale, making it difficult to discover spatial differences and patterns of carbon emissions within the region, which hinders the formulation and implementation of regional emission reduction policies^27,28^.

In view of the above issues, this article uses a long-term sequence nighttime light dataset combining corrected NPP-VIIRS and DMSP/OLS data. Based on long-term datasets, a carbon emission estimation model is first constructed to spatialize carbon emissions at the grid level. Then, the temporal and spatial evolution characteristics of carbon emissions at the provincial scale, market scale, county scale and grid scale in the CCEZ from 2000 to 2020 are analyzed systematically, and the law of carbon emission migration and development is determined through long-term evolutionary characteristics of carbon emissions.

## Materials and methods

### Study area

The Chengdu-Chongqing Economic Zone (CCEZ) belongs to the first-tier city cluster in China, alongside more developed economic zones such as the Beijing-Tianjin-Hebei region and the Pearl River Delta^29^. Located in the Sichuan Basin, the CCEZ, with Chengdu and Chongqing as its core, is a strategic highland for inland economies. Its geographical location, natural resources, and positioning advantages are different from those of other city clusters in the eastern region; thus, its economic development mode and the resulting interrelationships are also different^30^. In recent years, the CCEZ has experienced rapid economic growth, accelerated industrialization and urbanization, increased energy consumption, and significantly increased carbon emissions. Based on these characteristics, exploring the characteristics of carbon emissions and the spatial correlation of carbon emissions among cities from a spatial perspective is beneficial for formulating tailored emission reduction policies based on regional characteristics, coordinating with adjacent regions to leverage their respective emission reduction advantages, and providing valuable experience and strategic references for China's and even the world’s low-carbon development strategies.

**Figure 1:**
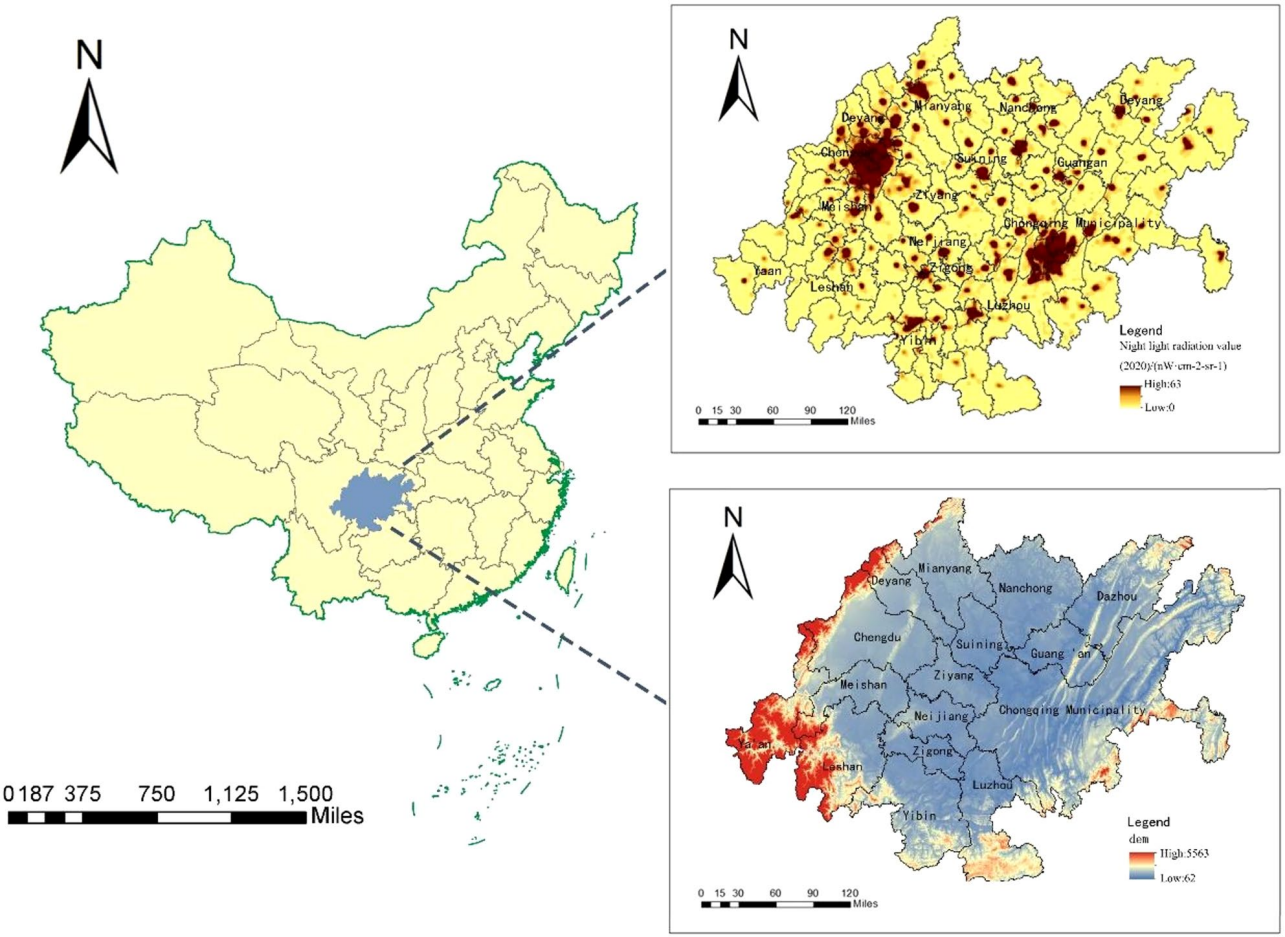
Geographic location and administrative division of the CCEZ.

The CCEZ includes 14 cities, such as Chengdu and Mianyang in Sichuan Province, as well as 29 counties in Chongqing, with a total area of 185,000 square kilometers. The data from the seventh National Census in 2020 show that the permanent population in the CCEZ was 97.58 million, accounting for 6.91% of the national population, and the regional GDP was 6602.9 billion yuan, accounting for 6.51% of the national GDP. In this study, the CCEZ, which contains 143 counties, was selected as the study area, as shown in Fig. 1.

### Data sources

The data sources for this study primarily fall into three categories: nighttime light remote sensing data from the CCEZ, county-scale carbon emission statistics, and data from the administrative division of the CCEZ. Table 1 provides detailed information on the data utilized.

**Table 1:** Data collection.

Data type	Data characteristics	Data sources
DMSP/OLS; NPP/VIIRS	DMSP/OLS are annual synthetic stabilized light data (2000–2013) with a spatial resolution of about 1 km; NPP/VIIRS data (2012–2020) have a spatial resolution of about 745 m	DMSP-OLS data from NGDC Data Center, NOAA website; NPP/VIIRS annual average light data from the academic department of the Colorado School of Mines’
County-scale carbon emission data	PSO-BP algorithm estimated CO_2_ emissions from 2735 counties in China from 1997–2017; linear regression method estimated CO_2_ emissions from 2018–2020	references^31^
County administrative boundary data	Provincial, municipal and district administrative boundaries 1:4 million	National basic geographic information system:

#### Stable nighttime light data

The DMSP/OLS (2000–2013) data were obtained from the official website of the National Geophysical Data Center (NGDC) of the National Oceanic and Atmospheric Administration (NOAA). The spatial resolution of the data is 1 km, and the pixel light value ranges from 0 to 63. The annual average light data of NPP/VIIRS (2012–2020) were obtained from the academic department of the Colorado School of Mines, with a spatial resolution of approximately 745 m. Due to the problem of pixel saturation and discontinuity between images obtained by different sensors, some researchers have carried out a series of corrections on DMSP/OLS and NPP/VIIRS data. In her study, Wu, Yizhen^31^ first employed a quadratic model along with the “pseudoinvariant pixel” method to rectify the DMSP-OLS data, aiming to alleviate discontinuities. Subsequently, she utilized an exponential smoothing model to forecast and fill in missing SNPP-VIIRS data, ensuring data continuity, and further removed outliers and noise from annual datasets. Subsequently, Wu employed a sigmoid model to generate improved SDMSP-OLS data, which more closely resembled the original DMSP-OLS data. These improved datasets were then merged with the calibrated DMSP-OLS data, forming enhanced DMSP-OLS-like data, thereby augmenting the data availability. Finally, qualitative and quantitative comparisons revealed robust linear correlations, indicating the enhanced ability to assess socioeconomic development and human activities effectively. This paper uses corrected nighttime light images of the Chengdu–Chongqing economic circle from 2000 to 2020.

#### County-scale carbon emissions data

These data are sourced from the literature^32^, where the particle swarm optimization-backpropagation (PSO-BP) algorithm was used to scale DMSP/OLS and NPP/VIIRS satellite images and estimate CO_2_ emissions for 2735 counties in China from 1997 to 2017. Based on the estimated CO_2_ emissions from 1997–2017, linear interpolation was used to calculate the CO_2_ emissions for 2018–2020. For example, the value for a certain county in 2018 is calculated as 2 times the value for that county in 2017 minus the value for that county in 2016.

#### County-scale administrative boundary data

These data are obtained from the National Basic Geographic Information System website (https://www.ngcc.cn/ngcc/) and provide administrative boundary data for provinces, prefecture-level cities, and counties in China at a scale of 1:4 million. This study utilized data for 143 counties, including 38 counties in Chongqing and 14 cities in Chengdu, Sichuan Province.

### Research methods

**Figure 2:**
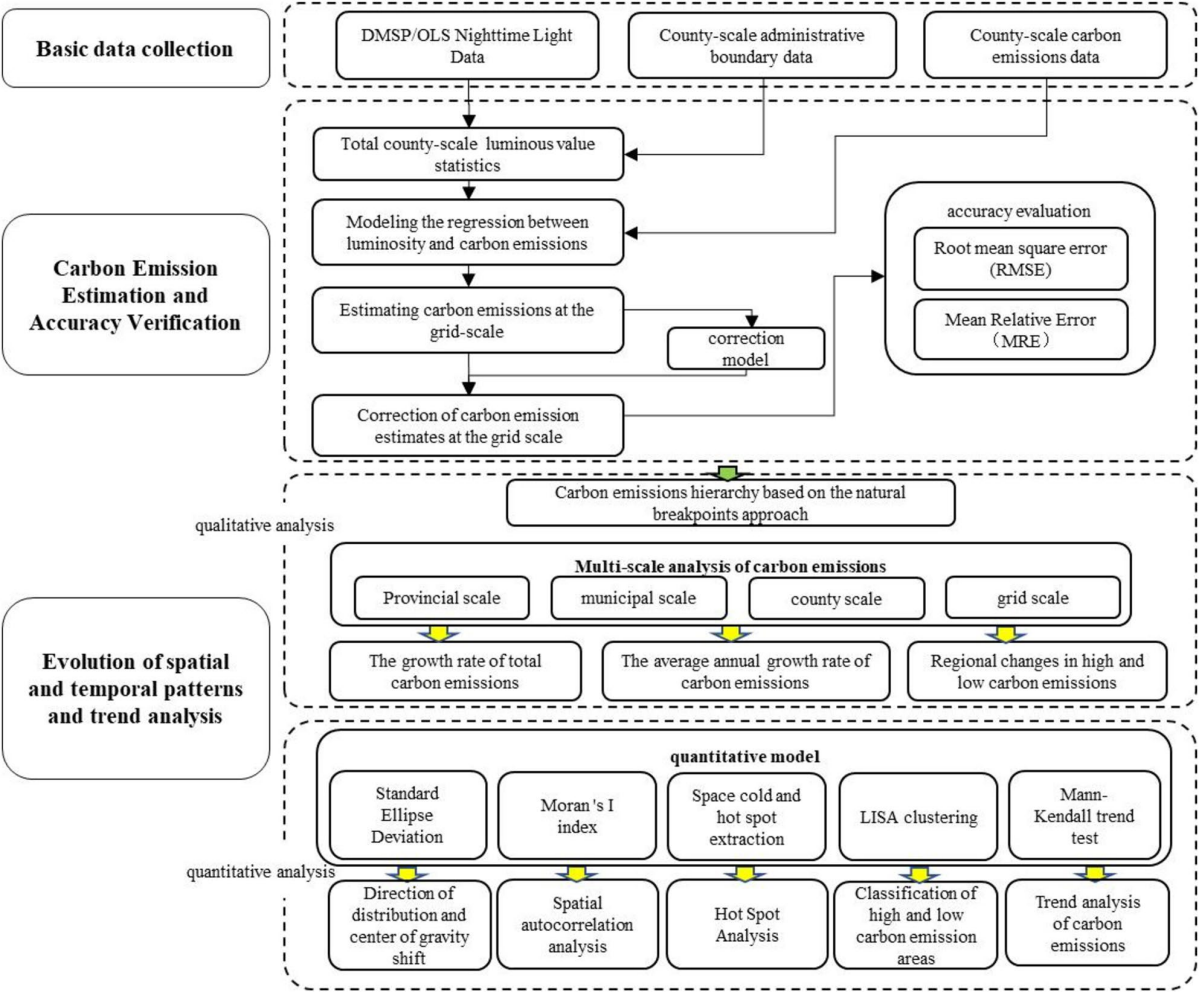
Overall architecture of this study.

To systematically analyze the spatiotemporal evolution characteristics of carbon emissions in the CCEZ from 2000 to 2020 at multiple scales and to discover the migration and development laws of carbon emissions in the CCEZ through long-term carbon emission evolution characteristics, this study adopts the research architecture shown in Fig. 2. First, carbon emission estimation and accuracy verification are conducted to establish an effective estimation model with a small root mean square error (RMSE) and mean relative error (MRE). Qualitative analysis is conducted on the spatiotemporal evolution of carbon emissions at the provincial, city, county, and grid scales. Finally, quantitative analysis is conducted on the spatiotemporal evolution of the regional patterns using methods such as standard deviation ellipse, global Moran’s I index, spatial hotspot and coldspot extraction, LISA clustering, and the Mann‒Kendall trend test.

#### Verification of carbon emission estimation and accuracy

##### Carbon emission estimation model

When constructing the carbon emission estimation fitting model, it is assumed that there is a good correlation between the total brightness of lights and the total carbon emissions. Based on the calibrated nighttime light data from 2000 to 2020, the relationship between the total value of nighttime lights and the corresponding carbon emissions in the CCEZ is established. Considering the accuracy issue of downscaling to grid units, a regression equation without intercepts is adopted to obtain the estimation model as follows:

1 $$M_{i} = a * DN_{i}^{3} + b * DN_{i}^{2} + c * DN_{i}$$

where E_i_ represents the estimation value of CO_2_ for grid unit i; DN_i_ represents the total nighttime light value of grid unit i; and a, b, and c are regression parameters.

##### Correct the grid estimation value

The initial carbon emission data E_s(n)_ for the CCEZ in the nth year can be obtained by calculating the carbon emission values reflected by nighttime lights in each grid unit using the carbon emission estimation fitting equation shown in Formula 1. Due to the presence of regression function errors, both E_s(n)_ and the actual carbon emission value E_r(n)_ have deviations. Therefore, the proportional coefficient M_n_ between the actual value and the estimated value of carbon emissions in the nth year of the CCEZ is constructed. The formula is as follows:

2 $$M_{n} = E_{r\left( n \right)} / E_{s\left( n \right)}$$

where M_n_ represents the proportional coefficient between the actual carbon emission value and the estimation value in the nth year of the CCEZ; E_r(n)_ represents the actual carbon emission value calculated from statistical data in the nth year of the CCEZ; and E_s(n)_ represents the estimation value obtained from the carbon emission estimation model in the nth year.

By multiplying the estimated carbon emissions in the nth year of the CCEZ by the proportional coefficient between the actual carbon emission value and the estimated value, the carbon emissions distributed on the kth grid unit in the nth year of the CCEZ are obtained. The calculation method is as follows:

3 $$E_{x\left( n \right)k} = E_{s\left( n \right)k} * M_{n}$$

where E_x(n)k_ represents the carbon emission distribution on the kth grid unit in the CCEZ in the nth year and E_s(n)k_ represents the carbon emission value reflected by nighttime lights on the kth grid unit.

##### Accuracy evaluation index

The root mean square error (RMSE) and mean relative error (MRE) are both indicators used to measure the predictive ability of regression models^33^. They measure the simulated ability of the model by calculating the difference between the actual values and the estimated value. The RMSE is the square root of the mean square error, and its formula is:

4 $$RMSE = \sqrt {\frac{1}{n}\mathop \sum \limits_{i = 1}^{n} \left( {Y_{i} - \widehat{{Y_{i} }}} \right)^{2} }$$

where n is the number of samples, Y_i_ is the actual value, and $\widehat{{Y_{i} }}{ }$ is the estimation value. The RMSE is sensitive to outliers and can reflect the distribution of simulated errors.

MRE refers to the mean absolute value of relative errors (relative errors are the ratio of errors to true values), and its formula is:

5 $$MRE = \frac{1}{n}\mathop \sum \limits_{i = 1}^{n} \left| {\frac{{Y_{i} - \widehat{{Y_{i} }}}}{{Y_{i} }}} \right|$$

where n is the sample size, Y_i_ is the true value, and $\widehat{{Y_{i} }}{ }$ is the s estimation value. MRE can reflect the magnitude of relative errors but cannot reflect the magnitude of absolute errors.

#### Qualitative analysis

A qualitative analysis of the spatiotemporal pattern evolution of carbon emissions at the provincial, municipal, county, and grid levels was conducted at multiple scales. First, the carbon emissions at different scales were classified into five levels using the natural breakpoint method. Levels 1–5 represent low carbon emissions, relatively low carbon emissions, moderate carbon emissions, relatively high carbon emissions, and high carbon emissions, respectively. The natural breakpoint method determines the classification of data based on the distribution characteristics of the data. It divides the data into a group of relatively uniform categories by calculating the differences between the data. Unlike the equal interval method and the equal proportion method, the natural breakpoint method does not rely on preset intervals or proportions but rather classifies the data based on its own characteristics. Based on the classification results using the natural breakpoint method, we analyzed the following aspects:

1. Growth rate of total carbon emissions

The growth rate of total carbon emissions in the CCEZ at multiple scales is calculated by dividing the carbon emissions in 2020 by the emissions in 2000 for the corresponding provinces, municipalities, and counties. By horizontally comparing different provinces, municipalities, and counties, we can determine the magnitude of their growth rates. This analysis can also explore the relationship between carbon emissions and economic development in different provinces, municipalities, and counties.

2. Annual average growth rate of carbon emissions

The annual average growth rate, also known as the compound growth rate, is a statistical concept that refers to the average rate of growth per year over a certain period of time. It is commonly used in population forecasts and can be calculated using the following formula:

6 $$Rn = ((C/Cn)^{ \wedge } (1/(n - 1)) - 1) \times 100\%$$

Here, R_n_ is the growth rate of the data in the nth year, C is the current period data, C_n_ is the data from the previous n years, and n is the number of years.

By calculating the average annual growth rate of carbon emissions in the CCEZ from 2000 to 2020, the trend of carbon emissions and the evaluation of carbon emission efficiency can be revealed.

3. Changes in high- and low-carbon emission regions

Based on the locations of low carbon emission, relatively low carbon emission, moderate carbon emission, relatively high carbon emission, and high carbon emission regions in the CCEZ, the changes in high and low carbon emission regions over time in spatial terms are compared to provide a basis for formulating more scientific and rational regional carbon emission control policies.

#### Quantitative analysis

To further quantify the spatiotemporal evolution patterns and trends of carbon emissions in the CCEZ from 2000 to 2020 and to further validate the results of qualitative analysis, the quantitative analysis section employs methods such as standard deviation ellipse, global Moran’s I index, spatial hotspot extraction, LISA clustering, and the Mann‒Kendall trend test model to analyze the distribution direction and centroid migration of carbon emissions, spatial autocorrelation relationships, spatial hotspot distribution, agglomeration types of high- and low-carbon emission areas, and the evolution trend of carbon emissions.

1. Standard deviation ellipse method

The standard deviation ellipse, also known as the directional distribution, was originally proposed by D. Welty Lefever, a sociology professor at the University of Southern California, as a classic algorithm for analyzing the direction and distribution of points. It can measure the directionality of the element distribution. This method uses the mean center of the elements as the origin and the standard deviations of the x and y coordinates as the axes to create an elliptical shape^34^. The three elements of the standard deviation ellipse are the rotation angle, major axis, and minor axis. The major axis represents the direction with more spatial distribution, while the minor axis represents the direction with less spatial distribution^35^. The calculation method is as follows:

a Calculate the mean. For a multivariate dataset with n samples, the mean vector is:
  7 $$\overline{x} = \frac{1}{n}\mathop \sum \limits_{i = 1}^{n} x_{i}$$
  where x_i_ represents the i-th sample.
b Calculate the covariance matrix. For a multivariate dataset with n samples, the covariance matrix is:
  8 $$S = \frac{1}{n - 1}\mathop \sum \limits_{i = 1}^{n} (x_{i} - \overline{x})\left( {x_{i} - \overline{x}} \right)^{T}$$
  where $\left( {x_{i} - \overline{x}} \right)\left( {x_{i} - \overline{x}} \right)^{T}$ represents the outer product, S is a p × p matrix, and p is the number of variables.
c Calculate the eigenvalues and eigenvectors. For the covariance matrix S, calculate its eigenvalues λ_1_, λ_2_,…, λ_p_ and corresponding eigenvectors v_1_, v_2_…, v_p_.where F represents the critical value of the statistical distribution. In general, F is 2.4477, which means that at the 95% confidence level, the data should be distributed within the standard deviation ellipse^36^.
d Calculate the lengths of the major and minor axes of the standard deviation ellipse. The length of the major axis is 2, and the length of the minor axis is 2.$\sqrt {\lambda_{1} F} \sqrt {\lambda_{2} F}.$ where F represents the critical value of the statistical distribution. In general, F is 2.4477, which means that at the 95% confidence level, the data should be distributed within the standard deviation ellipse^36^.

$\overline{x}{ }$ represents the average center point of the ellipse, which is the mean of the dataset, while its direction is defined by the largest and second-largest eigenvalues^32^. If the two eigenvalues are equal, the ellipse can be in any direction^33^. In this study, the average center point of the standard deviation ellipse of carbon emissions in the CCEZ from 2000 to 2020 is calculated, and the points are connected in chronological order to determine the direction of carbon emission centroid migration^36,37^.

2. Global Moran’s I index

Global spatial autocorrelation can reflect the overall characteristics of spatial correlation^36^. The overall spatial differences in carbon emissions can be described by the global Moran’s I index, which is calculated as follows:

9 $$I = \frac{{n\mathop \sum \nolimits_{i = 1}^{n} \mathop \sum \nolimits_{j = 1}^{n} W_{ij} (x_{i} - \overline{x})\left( {x_{j - } \overline{x}} \right)}}{{\mathop \sum \nolimits_{i = 1}^{n} \mathop \sum \nolimits_{j = 1}^{n} (x_{i} - \overline{x})^{2} \mathop \sum \nolimits_{j = 1}^{n} w_{ij } }}$$

where n represents the number of administrative units; x_i_ and x_j_ represent the carbon emissions of administrative units i and j, respectively; $\overline{x}$ represents the average carbon emissions of the units; and $W_{ij}$ represents the spatial weight matrix between adjacent administrative units i and j. The range of values for i is within [− 1, 1], where a value closer to 1 indicates a more significant positive spatial correlation, and a value closer to − 1 indicates a more significant negative correlation.

3. Spatial hotspot analysis

Hotspot analysis can be used to calculate the Getis-Ord G_i_* statistic for each feature in a concentrated dataset. By obtaining the z score and p value, one can analyze whether the clustering characteristics of high and low values in the spatial distribution are significant. The working principle of this tool is to examine each feature in the neighboring feature environment. High-value features often easily attract attention, but they may not be statistically significant hotspots^38^. To be a statistically significant hotspot, a feature should have a high value and be surrounded by other features with high values. The local sum of a feature and its neighboring features is compared to the sum of all features,

When there is a significant difference between the local sum and the expected local sum, to the extent that it cannot be attributed to random chance, a statistically significant z score is generated. The Getis-Ord local statistics can be represented as:

10 $$G_{i}^{*} \frac{{\mathop \sum \nolimits_{j = 1}^{n} W_{i,j} X_{j} - \overline{X}\mathop \sum \nolimits_{j = 1}^{n} W_{i,j} }}{{S\sqrt {\frac{{\left[ {n\mathop \sum \nolimits_{j = 1}^{n} W_{i,j}^{2} - \left( {\mathop \sum \nolimits_{j = 1}^{n} W_{i,j} } \right)^{2} } \right]}}{n = 1}} }} =$$

where ${\text{ X}}_{{\text{j}}}$ is the attribute value of factor j, $W_{i,j}$ is the spatial weight between factors i and j, n is the total number of factors, and

11 $$\overline{X} = \frac{{\mathop \sum \nolimits_{j = 1}^{n} X_{j} }}{n}$$

12 $$S = \sqrt {\frac{{\mathop \sum \nolimits_{j = 1}^{n} X_{j}^{2} }}{n}}$$

The Gi* statistic returned for each factor in the dataset is the z score. For statistically significant positive z scores, the higher the z score is, the tighter the clustering of high values (hotspots). For statistically significant negative z scores, the lower the z score is, the tighter the clustering of low values (coldspots).

4. LISA clustering

By conducting LISA clustering tests, the degree of agglomeration between local units and neighboring units can be revealed, and specific regions of high-value clustering and low-value clustering can be identified^36^. The calculation formula is as follows:

13 $$I_{i} = \frac{{n\left( {X_{i} - \overline{X}} \right)\mathop \sum \nolimits_{j = 1}^{n} W_{ij} \left( {X_{j} - \overline{X}} \right)}}{{\mathop \sum \nolimits_{i = 1}^{n} \left( {X_{i} - \overline{X}} \right)^{2} }}$$

when ${\text{I}}_{{\text{i}}}$ > 0, it indicates positive spatial autocorrelation between adjacent regions; when ${\text{I}}_{{\text{i}}}$ < 0, it indicates negative spatial autocorrelation between adjacent regions.

5. Mann‒Kendall trend test

The Mann‒Kendall trend test is a nonparametric statistical method used to detect trend changes in time series. It does not require any assumptions about the data distribution, making it suitable for various types of data^39^. The hypothesis of the Mann‒Kendall test is as follows: the null hypothesis—there is no trend change in the time series—and the alternative hypothesis—there is a trend change in the time series. The basic idea is to examine the trend in the sequence by comparing the size relationship between each pair of data points and then determine the direction of the trend based on the positive or negative nature of the rank sum^40^. The calculation process of the Mann‒Kendall trend test includes the following steps:

a For a time series X with a length of n, calculate the n (n − 1)/2 variable relationship difference (g):
  14 $${\text{g}}_{{[{\text{i}},{\text{j}}]}} = {\text{X}}_{{[{\text{j}}]}} - {\text{X}}_{{[{\text{i}}]}} \left( {{\text{i}} < {\text{j}}} \right)$$
b Multiply the signs of all g[ i,j] to obtain S_gn_. That is:
  15 $${\text{S}}_{{{\text{gn}}}} = {\text{Prod}}\left( {{\text{sign}}\left( {{\text{g}}_{{[{\text{i}},{\text{j}}]}} } \right)} \right)$$
c Calculate the sum of the ranks of the absolute values of all g_[i,j]_. That is:
  16 $${\text{sum}}\left( {{\text{rank}}\left( {|{\text{g}}_{{[{\text{i}},{\text{j}}]}} |} \right)} \right)$$
d Calculate the z statistic of the standard normal distribution, that is:
  17 $${\text{S}}_{{{\text{gn}}}} * \left( {{\text{S}} - {1}} \right)/{\text{sqrt}}\left[ {\left( {{\text{n}} * \left( {{\text{n}} - {1}} \right) * \left( {{2} * {\text{n}} + {5}} \right)} \right)/{1}} \right]$$
e Based on the significance level, select the critical value for rejecting the null hypothesis. If the z value is smaller than the critical value, the null hypothesis is accepted; otherwise, the null hypothesis is rejected.

After determining the presence of a trend using the Mann‒Kendall test, Sen’s slope can be used to calculate the slope of the trend. Sen’s slope is a method for estimating the slope of a trend using median differences. Unlike linear regression, Sen’s slope is not affected by outliers, making it more suitable for data with outliers. The calculation method for Sen’s slope is as follows:

a For a time series X with a length of n, calculate the n(n − 1)/2 variable relationship difference g:
  18 $${\text{g}}_{{[{\text{i}},{\text{j}}]}} = {\text{X}}_{{[{\text{i}}]}} \left( {{\text{i}} < {\text{j}}} \right)$$
b Calculate the median of the absolute values of all g_[i,j]_. That is:
  19 $${\text{M}} = {\text{median}}(|{\text{g}}_{{[{\text{i}},{\text{j}}]}} |)$$
c Sen’s slope was calculated. That is:
  20 $${\text{S}} = {\text{M}}/{\text{k}}$$

where k = (n − 1)/2.

Sen’s slope represents the rate of change per unit time in a time series. A positive Sen’s slope indicates an upward trend in the time series, and a negative Sen’s slope indicates a downward trend in the time series.

## Results and analysis

### Accuracy evaluation

Table 2 shows the fitting equation results between the total nighttime light value and CO_2_ emissions in the CCEZ from 2000 to 2020. The R^2^ of the fitting equation is more than 80%, indicating a good fitting effect and valid results. To ensure the reliability of the estimated carbon emissions in the CCEZ, they are compared with the corresponding actual carbon emissions. The root mean square error (RMSE) and mean relative error (MRE) between the estimated values and the actual values from 2000 to 2020 are shown in Table 2. Table 3 shows that the RMSE ranges from 23.99 to 77.80 thousand tons, and the MRE of most years ranges from 3.73 to 10%, with no errors exceeding 15% for all years. Nighttime light data have good estimation accuracy for CO_2_ emissions and can be used to study the spatial and temporal evolution characteristics of CO_2_ emissions in the CCEZ.

**Table 2:** Fitting equation of the total nighttime light value and CO_2_ emissions from 2000 to 2020.

Year	Fitting equation	R^2^
2000	y = 10.237x^3^−236.61x^2^ + 735.33x	0.8080
2001	y = 300.67x^3^−393.98x^2^ + 663.32x	0.8175
2002	y = 196.37x^3^−311.69x^2^ + 621.83x	0.8380
2003	y = 302.16x^3^−472.92x^2^ + 702.93x	0.8544
2004	y = 213.92x^3^−357.55x^2^ + 707.81x	0.8610
2005	y = 250.43x^3^−436.64x^2^ + 809.9x	0.8885
2006	y = 254.82x^3^−497.06x^2^ + 842.52x	0.8872
2007	y = 186.01x^3^−379.85x^2^ + 826.93x	0.9031
2008	y = 114.66x^3^−281.21x^2^ + 844.86x	0.8862
2009	y = 29.539x^3^−142.85x^2^ + 833.85x	0.8447
2010	y = −11.668x^3^−60.193x^2^ + 727.41x	0.8534
2011	y = −7.4347x^3^−22.325x^2^ + 779.46x	0.9182
2012	y = 21.941x^3^−89.179x^2^ + 780.21x	0.9295
2013	y = 7.3671x^3^−58.01x^2^ + 650.47x	0.8994
2014	y = 6.483x^3^−73.737x^2^ + 593.55x	0.8575
2015	y = −16.026x^3^ + 7.2604x^2^ + 515.13x	0.8639
2016	y = −5.1062x^3^−26.716x^2^ + 501.44x	0.8745
2017	y = −13.868x^3^ + 41.566x^2^ + 379.25x	0.8746
2018	y = −11.492x^3^ + 38.784x^2^ + 353.6x	0.8280
2019	y = −10.394x^3^ + 37.062x^2^ + 339.44x	0.8086
2020	y = −17.706x^3^ + 62.239x^2^ + 384.96x	0.8127

**Table 3:** Accuracy test of carbon emissions estimation from 2000 to 2020.

Year	RMSE/10,000 tons	MRE/%
2000	23.99073	6.66
2001	27.03599	9.35
2002	30.68152	10.03
2003	47.08435	13.35
2004	41.98124	11.20
2005	52.35591	12.13
2006	77.63108	15.34
2007	61.93229	11.80
2008	56.0133	10.40
2009	40.32512	4.92
2010	49.5187	3.73
2011	58.05496	9.51
2012	32.43477	3.78
2013	36.40506	3.80
2014	72.98782	6.57
2015	62.2747	10.93
2016	77.80812	4.14
2017	39.71286	4.67
2018	34.17475	5.15
2019	38.25536	5.52
2020	57.20558	7.44

### Qualitative analysis

#### Provincial scale

**Figure 3:**
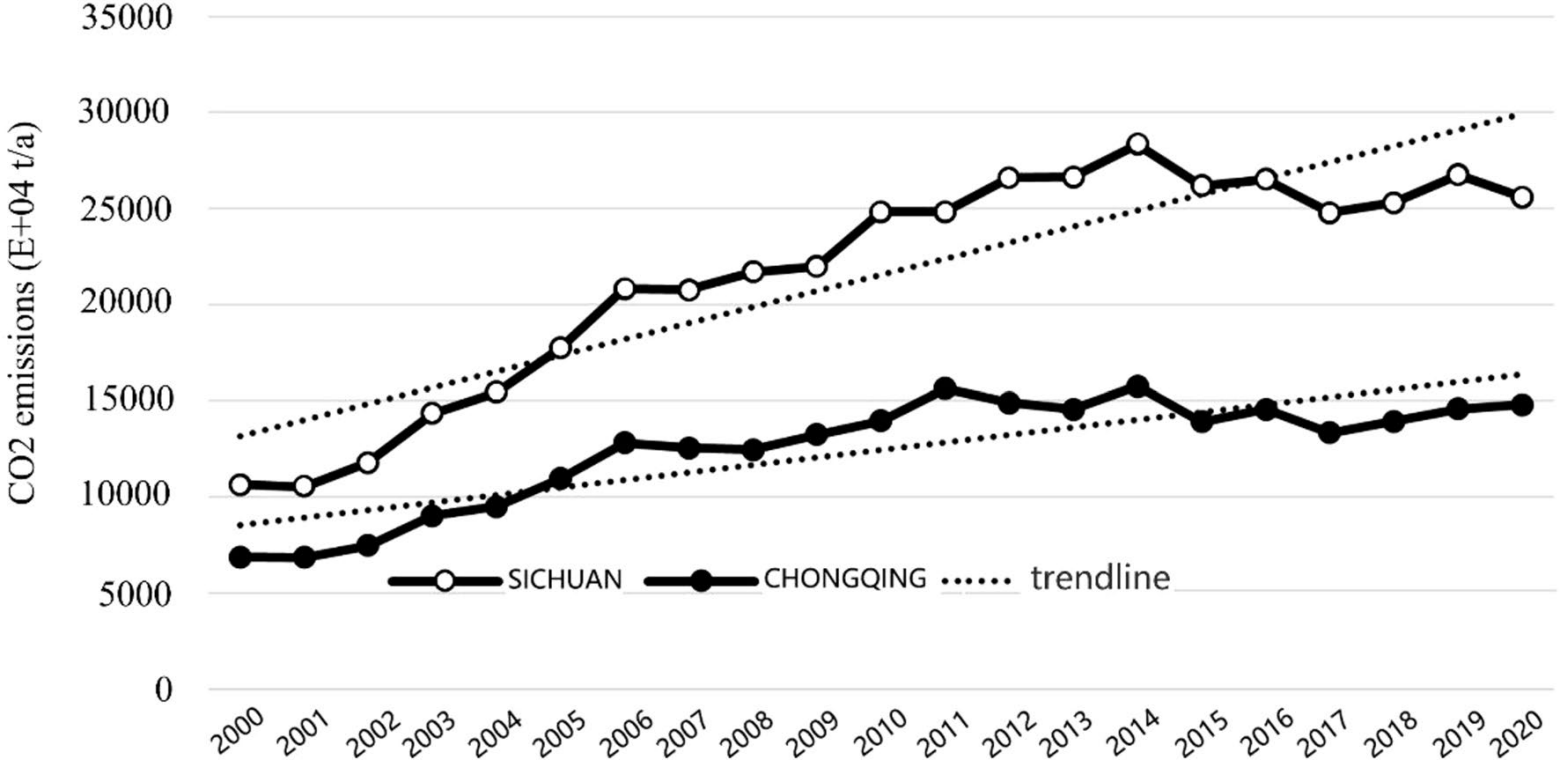
CO_2_ emissions in the CCEZ from 2000 to 2020.

Overall, carbon emissions in the CCEZ continued to increase from 2000 to 2020, an increase of 2.5 times. Chengdu and Chongqing have the highest carbon emissions in the CCEZ. Over the 21-year period at the provincial scale, the carbon emissions in Sichuan Province increased from 101.94 million tons in 2000 to 258.58 million tons in 2020, an increase of more than 2.53 times. Chongqing increased from 68.63 million tons to 147.89 million tons, an increase of 2.15 times. Figure 3 shows the CO_2_ emissions in Sichuan Province and Chongqing from 2000 to 2020. The total carbon emissions in Sichuan Province and Chongqing Municipality increased rapidly from 2000 to 2006, with average annual growth rates of 11.99% and 4.95%, respectively. From 2006 to 2014, they showed a steady upward trend, with average annual growth rates of 3.59% and 2.32%, respectively. From 2014 to 2020, they showed a fluctuating and slow upward trend.

#### Municipal scale

**Figure 4:**
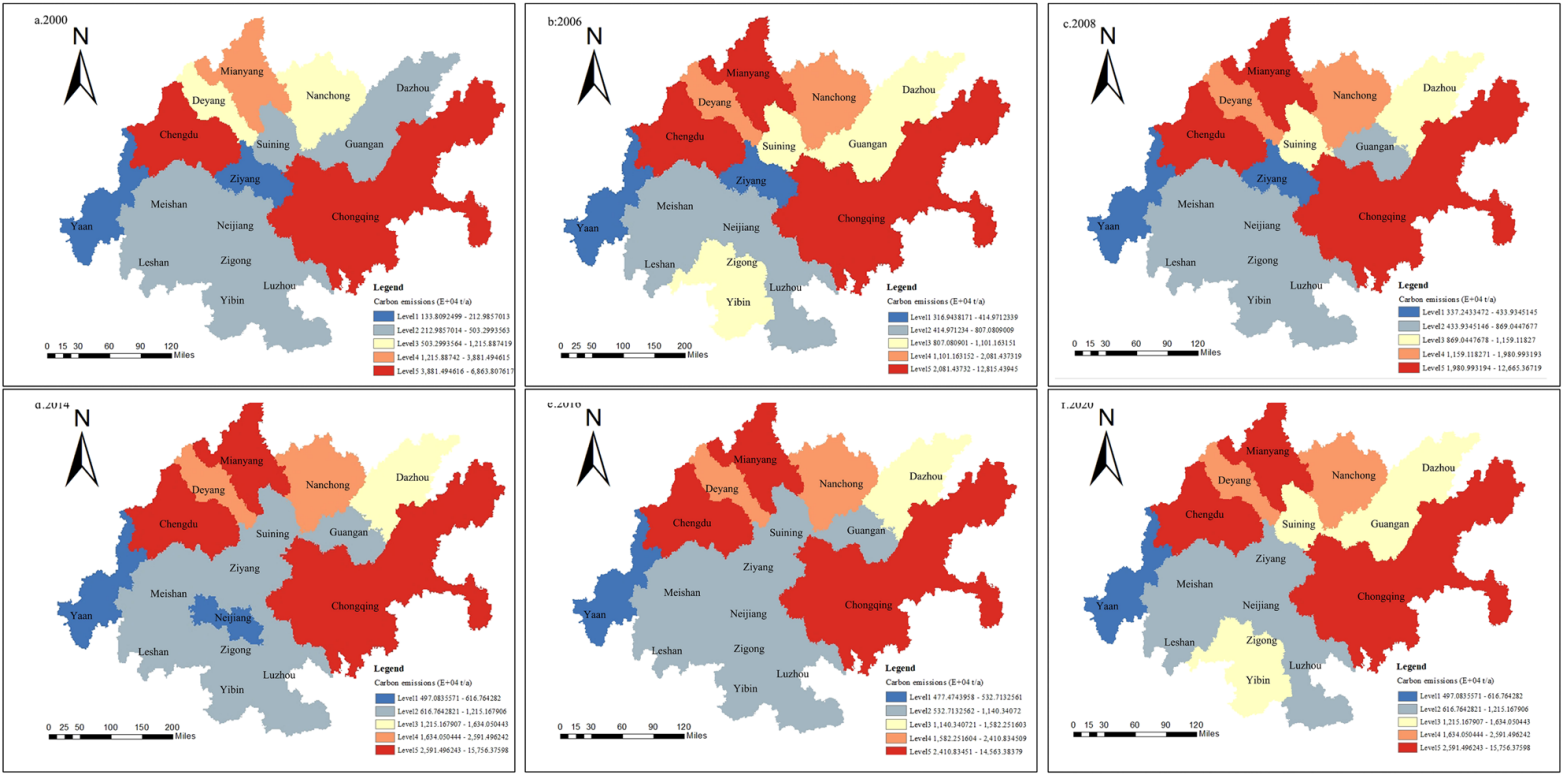
Characteristics of carbon emission evolution at the city level.

As shown in Figure. Figure 4 shows that the carbon emissions of Chengdu and Chongqing are significantly greater than those of other cities. With the passage of time, the number of lower and higher carbon emission areas in the Chengdu–Chongqing economic circle increases, and the higher carbon emission areas expand to the north. The lower carbon emission area tends to expand to the south.

In 2000, high carbon emission areas were distributed in Chengdu and Chongqing, higher carbon emission areas were distributed in Deyang city, and low carbon emission areas and lower carbon emission areas accounted for a large proportion. The overall carbon emissions show a pattern of more in the northeast and less in the southwest; from 2000 to 2006, high carbon emission areas and higher carbon emission areas increased. High carbon emission areas increased in Mianyang city, and higher carbon emission areas increased in Deyang city and Nanchong city. This may be due to the formal launch of regional planning in 2006, and put forward: “in the next 5 to 10 years, it is necessary to actively build the largest dual-core urban agglomeration in western China, with Chengdu and Chongqing as the center and central cities at all levels connecting and cooperating with each other”. This is the first time that the concept of the Chengdu–Chongqing economic zone has appeared in the report at the national level, and the cities have developed rapidly under the promotion of relevant policies. From 2006 to 2008, first, there was a reduction in total carbon emissions. It may be that the Wenchuan earthquake occurred in that year, which affected the economic development of the city in the Chengdu–Chongqing economic circle and reduced carbon emissions. Second, in terms of the regional distribution of high and low carbon emissions, the high carbon emission area and the higher carbon emission area remain unchanged, while the lower carbon emission area shows an expansion trend on the original basis, increasing Yibin and Guang’an. This may be due to the industrial restructuring and upgrading of the Guang’an and Yibin cities, which have transformed from traditional manufacturing with high energy consumption and high emissions to low-energy and low-emission industries such as high-end manufacturing and modern services, thus reducing carbon emissions. From 2008 to 2014, low-carbon emission areas continued to increase, increasing in Suining city. From 2014 to 2020, the growth trend of overall carbon emissions in the Chengdu–Chongqing economic circle was stable, the high carbon emission area and the higher carbon emission area remained unchanged, and the medium carbon emission zone increased in Guang’an, Suining and Yibin. In addition, Ya’an city and Leshan city are rich in forest resources, forest coverage is more than 50%, the proportion of secondary industry is small, and carbon emissions are low.

#### County scale

**Figure 5:**
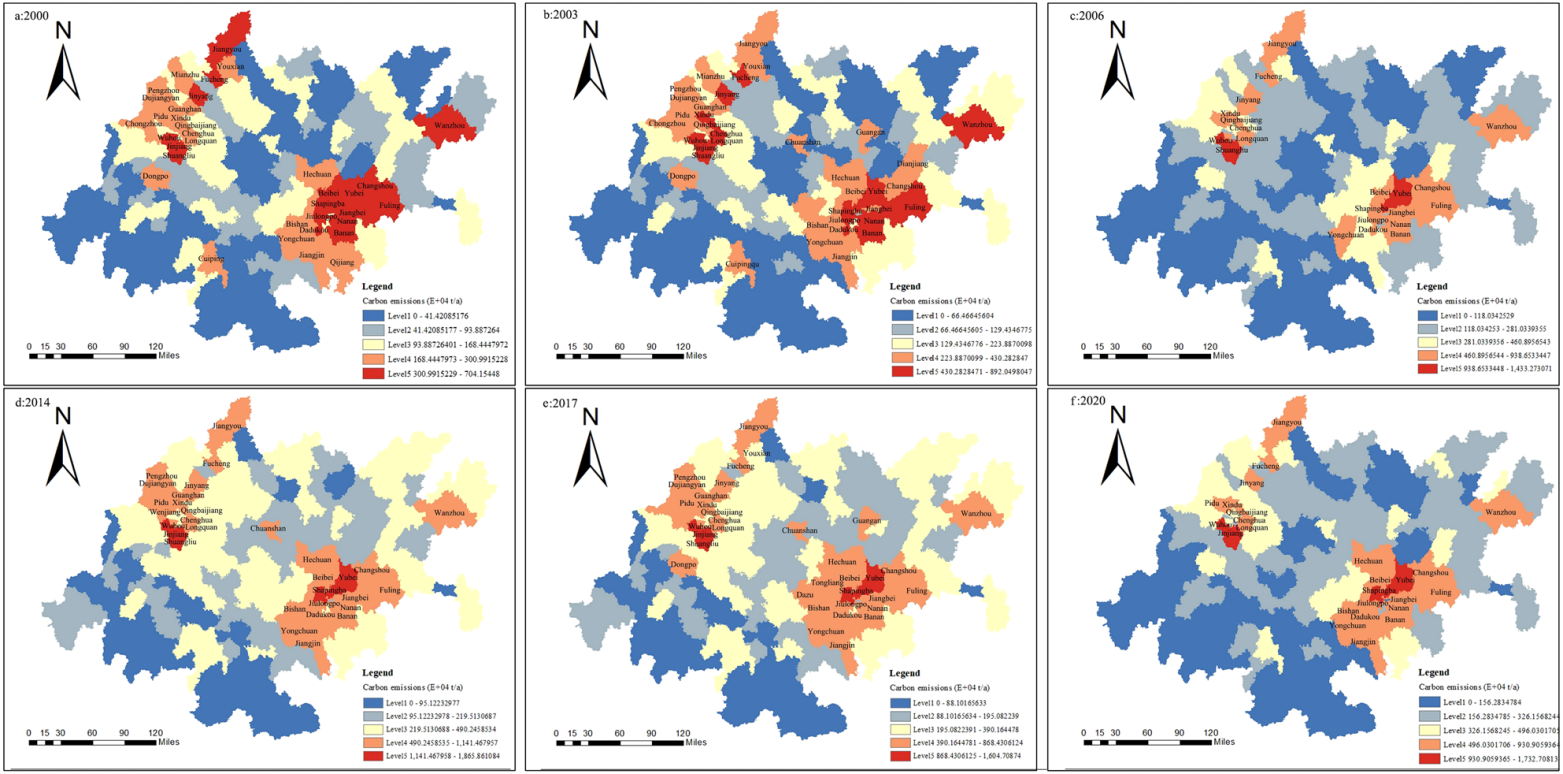
Evolutionary characteristics of carbon emissions at the county level.

At the county scale, based on the estimation results of carbon emissions, the carbon emissions of county-level administrative regions in the CCEZ are obtained through zoning statistics. Figure 5 shows the county-level carbon emissions in the CCEZ from 2000 to 2020. In terms of total carbon emissions, counties with high carbon emissions account for 1% to 6%, and these counties are mainly distributed in the Shuangliu District and Wuhou District of Chengdu, Yubei District and Tongliang district of Chongqing, etc.. Low carbon emission areas are concentrated in the peripheral cities of the Chengdu–Chongqing urban agglomeration and cities with lower industrialization levels around the Sichuan Basin, with low carbon emission counties accounting for 21% to 41%.

In terms of spatial structure, initially, a multicenter-periphery structure was observed. Several centers were distributed in the western, eastern, and northern parts of the CCEZ, with higher carbon emissions in the central counties and lower carbon emissions in the surrounding areas. This means that initially, the carbon emissions in the eastern, western, and northern regions were higher, while the central and southern regions had relatively lower carbon emissions. Over time, the high carbon emission areas in the northern part decreased, and a dual-core-periphery spatial structure with higher carbon emissions in the eastern and western core areas and lower carbon emissions in the surrounding areas began to form.

In terms of changes in the regions with high and low carbon emissions, in 2000, the counties with high carbon emissions were mainly distributed in Wuhou District, Jinjiang District, Shuangliu District of Chengdu, Fucheng District and Jiangyou of Mianyang, Jingyang District of Deyang, Yubei District, Shapingba District, and Banan District of Chongqing. The counties with relatively high carbon emissions were mainly concentrated in Chenghua District, Xindu District, Pixian District of Chengdu, Youxian District of Mianyang, Yongchuan District, Jiangjin District, and Hechuan District of Chongqing. In 2003, the carbon emissions in most counties within the CCEZ increased, and the counties with high carbon emissions added to Xindu District, Qingbaijiang, Chenghua District, and Longquanyi District of Chengdu. The counties with relatively high carbon emissions included the Chuanshan District of Suining, Guang’an District of Guang’an, Dazu District, and Dianjiang County of Chongqing. In 2006, carbon emissions showed a stable growth trend and began to change from a multicenter to a dual-core pattern. The main manifestation was that the distribution of carbon emissions expanded from the core areas of Jinjiang District and Shuangliu District of Chengdu and Yubei District of Chongqing. In 2014, carbon emissions showed a significant growth trend, mainly manifested by the expansion of the region with medium carbon emissions in the central part of the CCEZ. In 2017, due to the implementation of carbon emission reduction policies, the distribution of carbon emissions in the region decreased, which was mainly reflected in the reduction in the number of counties with high carbon emissions and relatively high carbon emissions in the central region. In 2020, the total carbon emissions continued to increase steadily, while those of the counties with high carbon emissions and relatively high carbon emissions remained unchanged. Overall, the main urban areas of Chengdu and Chongqing have large total carbon emissions, but the growth rate tends to be stable, while the surrounding counties, driven by their development, have a faster growth rate of carbon emissions.

#### Grid scale

**Figure 6:**
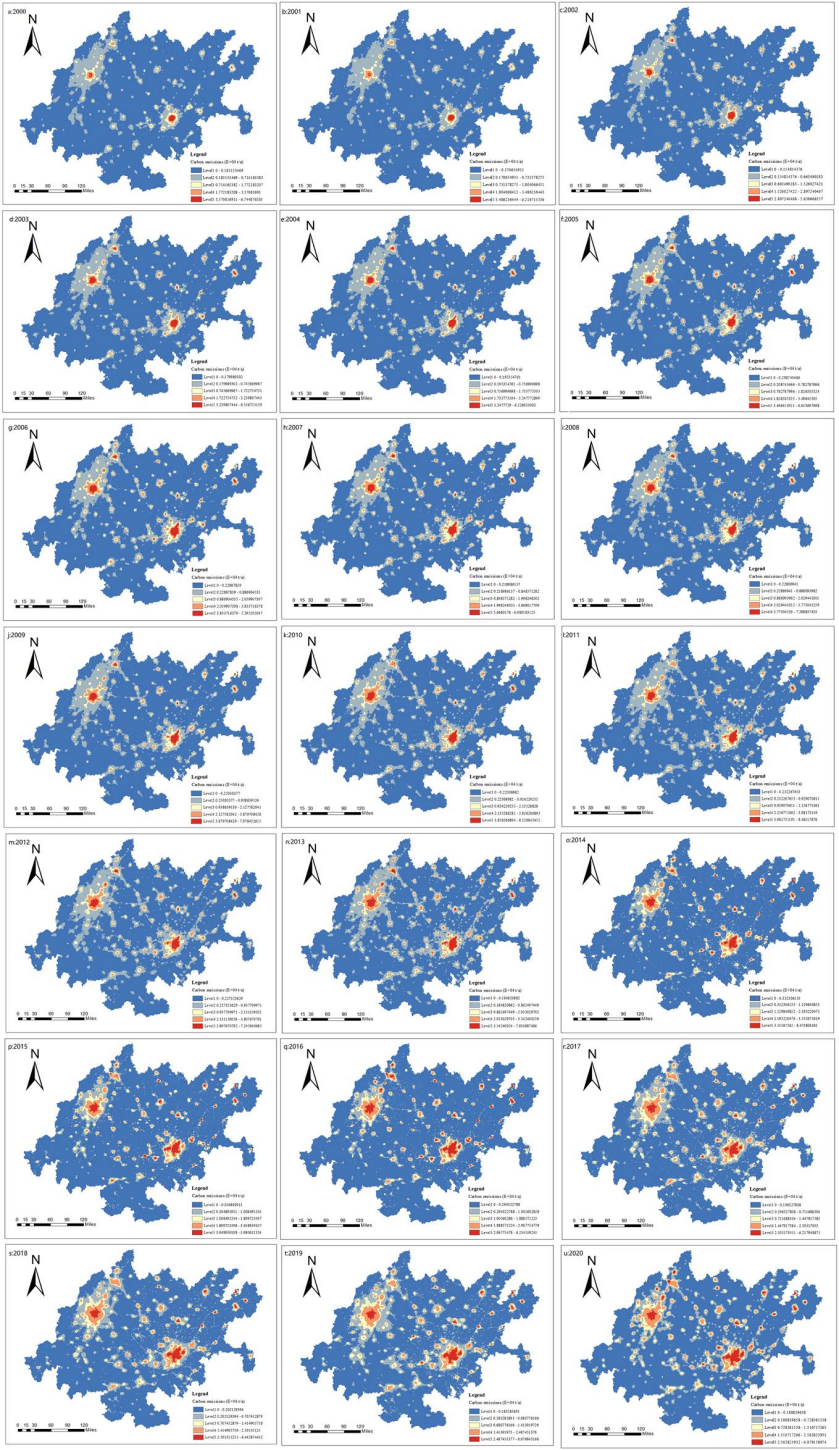
Evolutionary characteristics of carbon emissions at the grid scale.

Figure 6 shows the spatial distribution of carbon emissions at a 1 km × 1 km resolution from 2000 to 2020, obtained through estimation and inversion at the county scale. At the grid scale, the high carbon emission areas in the CCEZ from 2000 to 2020 exhibit a multicore distribution pattern, evolving into two main cores in Chengdu and Chongqing, gradually expanding radially outward, and ultimately forming a connected pattern of high carbon emission core areas. Through macroscopic analysis of the overall layout and municipal-scale positioning of the CCEZ, as well as analysis of carbon emission distribution at the county and grid levels, it was found that there are certain differences within the economic zone. Describing this phenomenon at multiple scales can provide an effective reference for precise emission reduction positioning.

### Quantitative analysis

#### Distribution direction and gravity center migration

**Figure 7:**
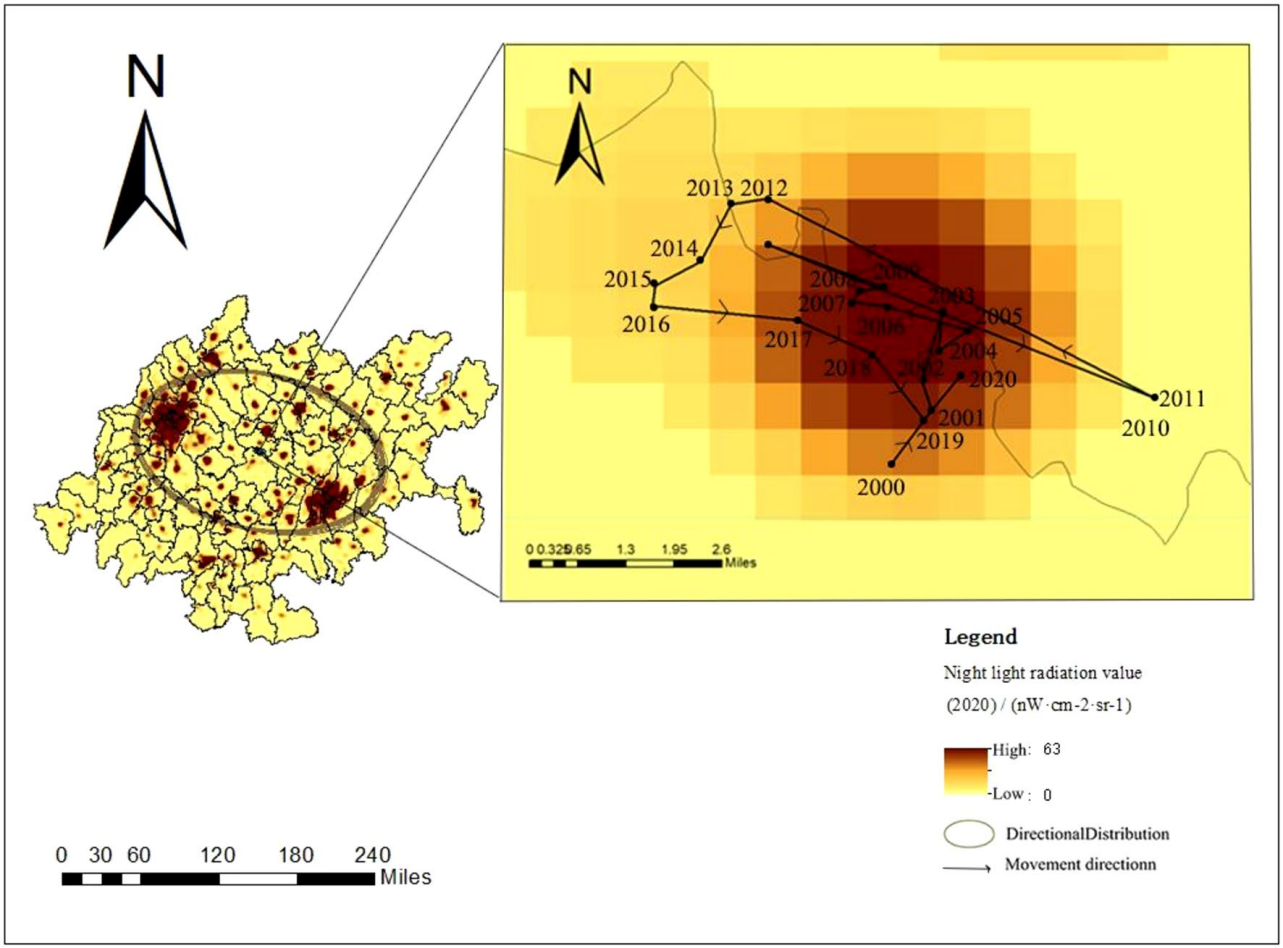
Direction of carbon emission center of gravity migration.

Figure 7 shows the results of carbon emissions analysis at the grid scale from 2000 to 2020. We found that the center of gravity of carbon emissions in the CCEZ moved northwestward from 2011 to 2016 and then started to move southeastward from 2016, shifting from a direction closer to Chengdu to a direction closer to Chongqing.

Combined with the data on the proportion of secondary industry in the regional GDP, from 2011 to 2016, the regional GDP of Chongqing increased from 55,462,990 to 78,945,626 CNY, representing a growth rate of approximately 1.42 times. The regional GDP of Chengdu increased from 31,462,516 to 52,332,004 yuan, representing a growth rate of approximately 1.66 times. The growth rate of the secondary industry’s GDP in Chengdu exceeded that in Chongqing, which is consistent with the conclusion that the center of carbon emissions shifted from Chongqing to Chengdu from 2011 to 2016. From 2016 to 2020, the regional GDP of Chongqing increased from 78,945,626 to 99,911,988 yuan, a growth rate of approximately 1.27 times. The regional GDP of Chengdu increased from 52,332,004 to 54,196,303 yuan, representing a growth rate of approximately 1.04 times. The growth rate of the secondary industry’s GDP in Chongqing exceeded that in Chengdu, which is consistent with the conclusion that the center of carbon emissions shifted from Chengdu to Chongqing after 2016 (Fig. 7).

In addition, high carbon emission areas have expanded outward from the two core cities, with the areas surrounding Chengdu showing the most significant high carbon emission. The carbon emissions in the cities adjacent to Chengdu and the central area of Chongqing increased significantly.

#### Spatial autocorrelation analysis

**Figure 8:**
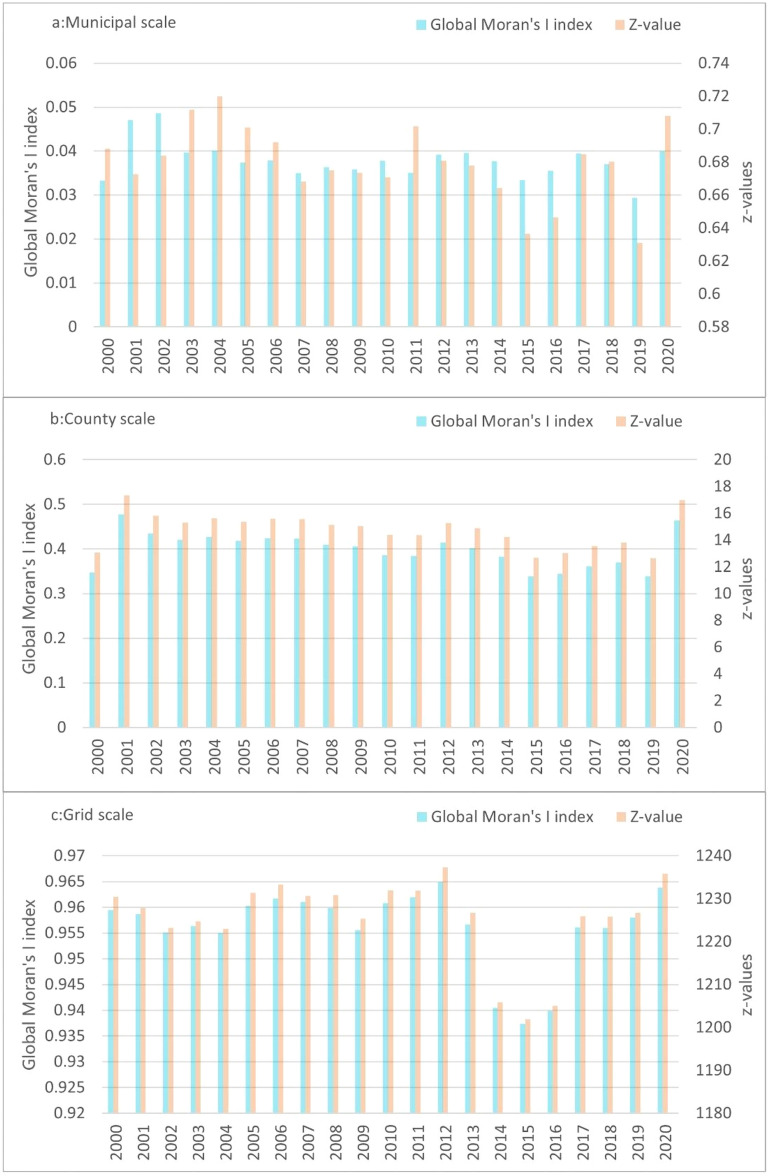
Multiscale spatial autocorrelation analysis.

Figure 8 shows the results of the spatial correlation measurements of carbon emissions at different scales in the CCEZ using the global Moran’s I index and corresponding z values. At the municipal scale, the global Moran’s I index for the CCEZ from 2000 to 2020 is positive, indicating the presence of spatial autocorrelation, but the autocorrelation may not be very strong. For example, in 2020, the z score is 0.688135, and in 2020, the z score is 0.707981, indicating some statistical significance. However, the p values are 0.491368 and 0.478957, respectively, indicating that the results are not statistically significant. The municipal-scale analysis is conducted on a larger spatial scale, focusing on the average situation of municipal-scale administrative units. At this scale, the spatial autocorrelation of carbon emissions in the CCEZ is weak. This may be due to differences in industrial structure, energy structure, population distribution, and other factors among different cities. These factors may affect the spatial distribution and correlation of carbon emissions.

At the county and grid scales, the global Moran’s I index of the CCEZ from 2000 to 2020 is positive and close to 1, indicating a strong positive spatial correlation in carbon emissions. This may be due to the close economic and geographical connections between cities in the Chengdu-Chongqing region, resulting in similar distribution patterns of carbon emissions. The z scores are much greater than the critical value of 1.96 for a standard normal distribution, indicating that this spatial autocorrelation is statistically significant at a level of 0.05 and not the result of random variation. The p values are all less than 0.05, indicating that this spatial autocorrelation is highly significant and credible.

Second, in terms of the changes in the global Moran’s I index from 2000 to 2020, the index shows an overall increasing trend over time. During the period from 2000 to 2021, the global Moran’s I value at the county level increased from 0.3471 to 0.4643, and the global Moran’s I value at the grid level increased from 0.9594 to 0.9638. This indicates that the spatial correlation of carbon emissions in the CCEZ is strengthened. At the county scale, carbon emissions show an agglomeration state, and the same applies at the grid scale, meaning that counties with higher carbon emissions are also surrounded by other counties with higher carbon emissions.

Analysis at the county and grid scales is conducted within a smaller spatial range, which can capture more detailed spatial information. At these two scales, there is a strong spatial autocorrelation in carbon emissions in the CCEZ, possibly due to significant geographical, economic, and climatic connections between counties in the region. In addition, spatial autocorrelation analysis alone cannot reveal the clustering status of carbon emissions at the county scale within the CCEZ. Therefore, further clustering is needed to explore the variations in carbon emissions at the county scale and discover the underlying patterns, providing theoretical references for the formulation of precise emission reduction policies.

#### Hotspot analysis

**Figure 9:**
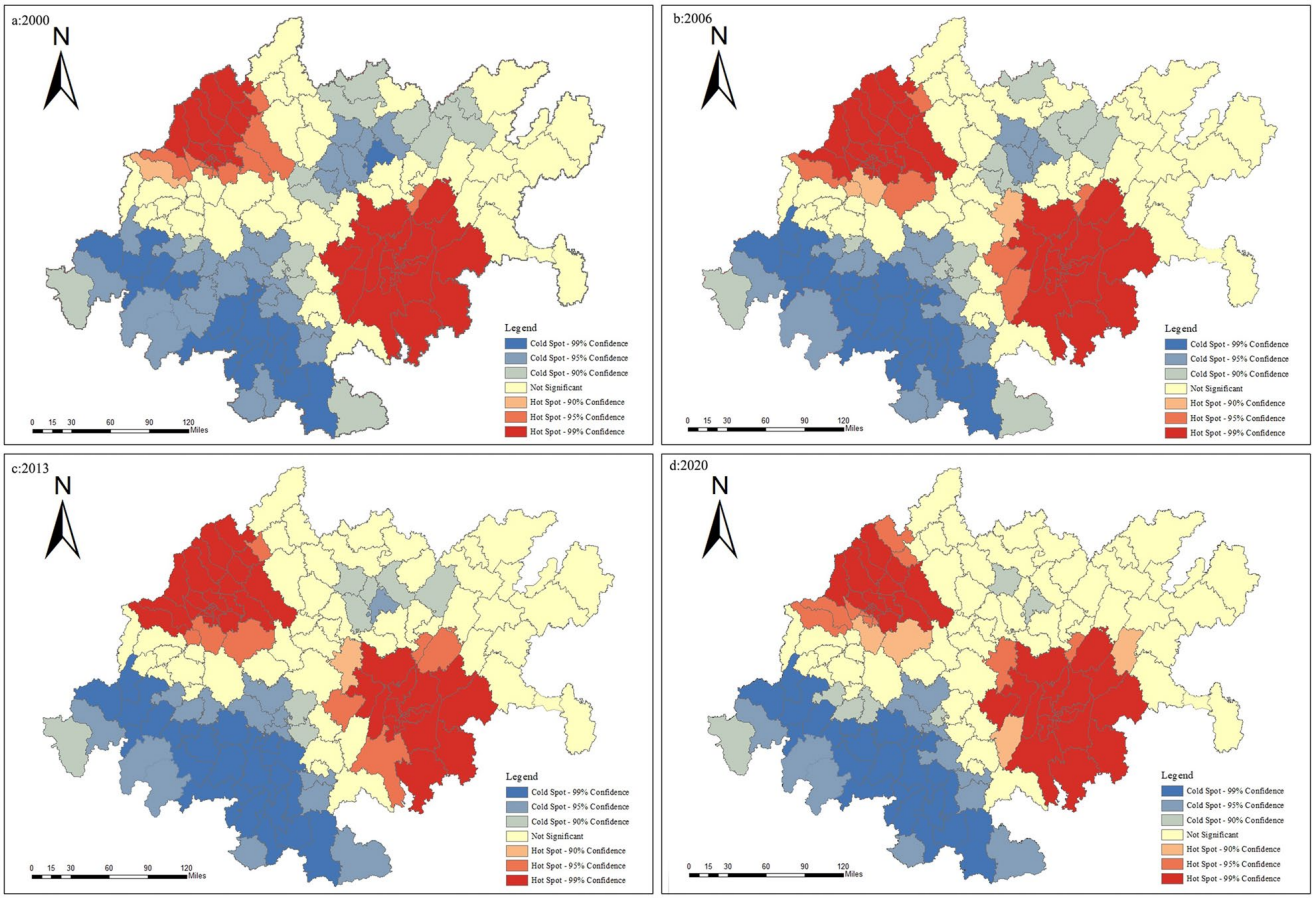
Analysis of hotspots at the county level.

Due to the significant autocorrelation relationship at the county and grid scales in the CCEZ, further hotspot analysis was conducted on carbon emissions. At the county scale, the distribution of carbon emission hotspots showed a significant dual-core outward diffusion trend, and the results are shown in Fig. 9. In terms of the distribution of carbon emission hotspots in the CCEZ, significant spatiotemporal differences were observed. The major hotspots were concentrated in Chenghua District and Pixian District of Chengdu city, Yubei District, Shapingba District, and Nan’an District of Chongqing city, indicating that these counties had high carbon emissions and exhibited an agglomeration state. The hotspots and subhotspots were located adjacent to the extreme hotspot areas distributed in Longquanyi District, Jintang District, Dayi County of Chengdu, and Huaying city of Chongqing, indicating the radiation effect of counties with high carbon emissions on their surrounding areas. The coldspots and subcoldspots were distributed in the peripheral cities of the Chengdu-Chongqing urban agglomeration and in cities with lower industrialization levels around the Sichuan Basin. The coldspot areas were mainly concentrated in Ya’an, Leshan, Yibin, and Nanchong, while the hotspot areas were concentrated in Chengdu, Deyang, and the main urban area of Chongqing, forming patchy extreme hotspot areas.

**Figure 10:**
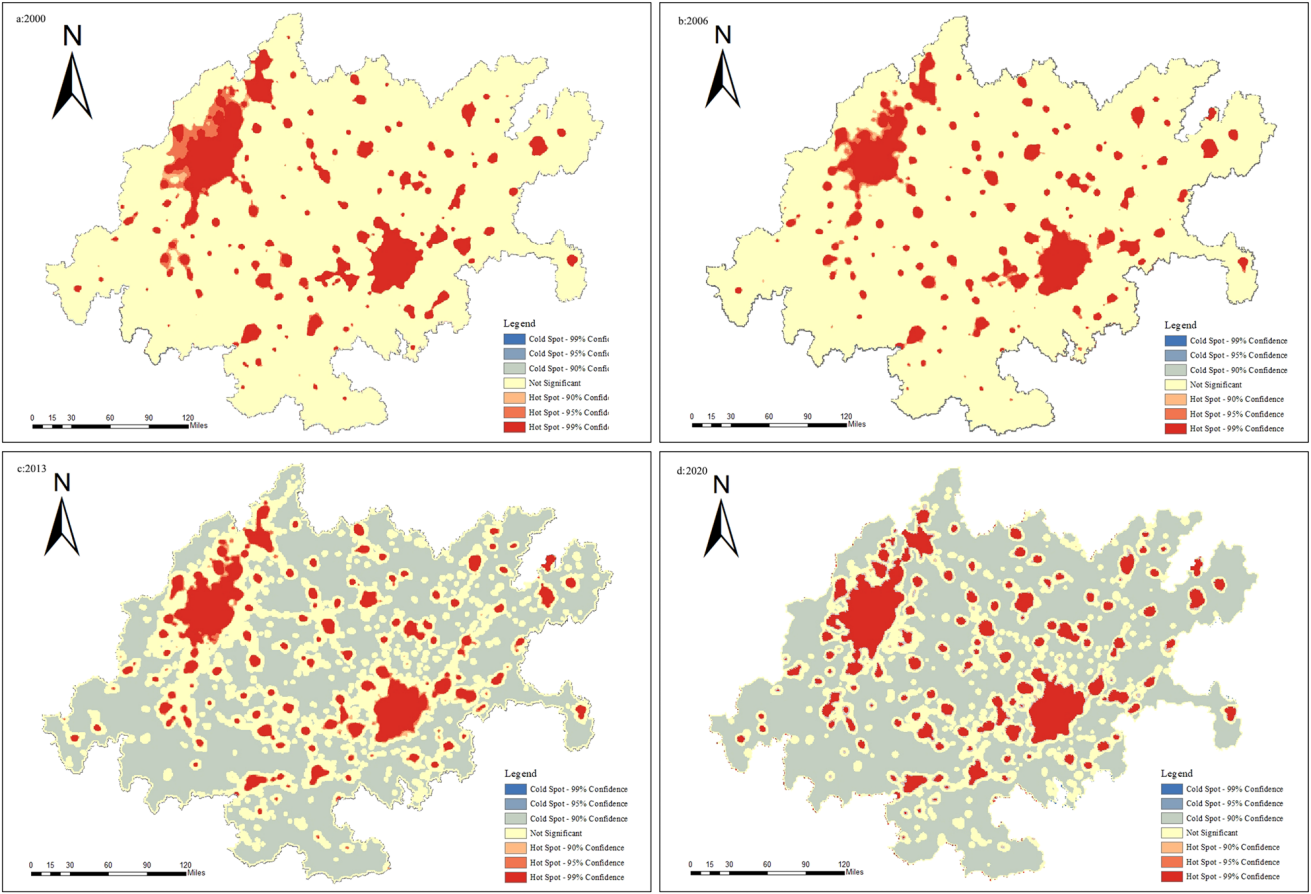
Grid-scale hotspot analysis.

At the grid scale, in 2000, the distribution of hotspots showed a scattered structure, with Chengdu and Chongqing being the main urban areas at the core. In 2013, the hotspot area structure changed, forming a belt radiating outward with Chengdu and Chongqing’s main urban areas as the core. In 2020, the hotspot area of carbon emissions continued to expand around the core area, and the results are shown in Fig. 10.

In conclusion, the spatial distribution of carbon emissions in the CCEZ is uneven. Chengdu and Chongqing’s main urban areas have large total carbon emissions, but the growth rate tends to be stable. The surrounding counties of Chengdu and Chongqing, driven by their development, have a faster growth rate of carbon emissions.

#### Lisa cluster analysis

**Figure 11:**
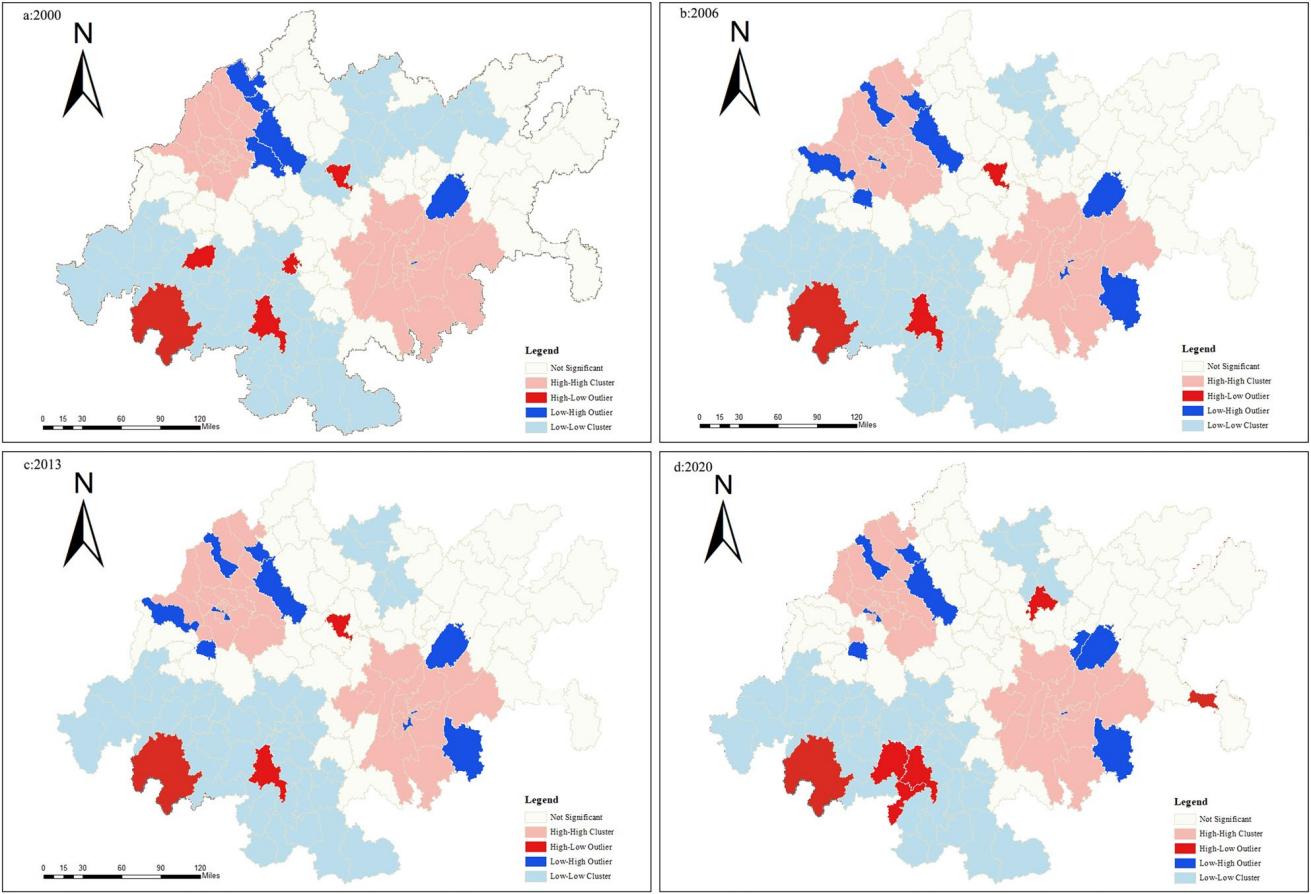
County-scale LISA clustering.

By using LISA clustering, the local spatial characteristics of carbon emissions at the county scale in the CCEZ are displayed. At the county scale, the distributions of counties with strong spatial autocorrelation from 2000 to 2020 are similar, and the results are shown in Fig. 11.

In 2000, there were 52 counties classified as LL-type, mainly distributed in cities such as Ya’an, Leshan, and Nanchong. There were 35 counties classified as HH-type, which were mainly distributed in most counties of Chengdu, Deyang, and Chongqing. Other counties were not significant regions. In 2006, there were 45 counties classified as LL-type, with decreases mainly in the northern region, including Dongxing District and Longchang in Neijiang, Anju District and Daying County in Suining, Tongchuan District, Dachuan District, and Qu County in Dazhou. The HH-type counties increased in Jintang County and Jianyang in Chengdu. The clustering areas in 2013 were similar to those in 2006, with a decrease in the number of LL-type counties in Yingshan County, Dazhou. The HH-type counties increased with Anzhou District in Mianyang. In 2020, the LL-type counties overlapped with those in 2013, and the HH-type counties decreased in Chongzhou District and Shuangliu District in Chengdu and Yongchuan District in Chongqing. Overall, the county-scale carbon emissions in the CCEZ show obvious clustering characteristics, and Chengdu, Deyang, and the municipal districts of Chongqing, which belong to the HH-type cluster, can be identified as precise emission reduction areas.

#### Carbon emission trend analysis

First, the carbon emission data at the grid scale for the years 2000–2020, for a total of 21 years, were transformed into panel data files. The files were then filtered to extract valid data and merged. Next, Sen’s slope algorithm was used to calculate the trend in carbon emissions for each grid from 2000 to 2020. The trend values were subjected to significance testing using the Mann‒Kendall trend test. A *p* value less than 0.05 indicated a reliable trend. After confirming the reliability of the calculation results, the trend calculation results were connected to the vector fishnet file, and the visualization results were finally output.

**Figure 12:**
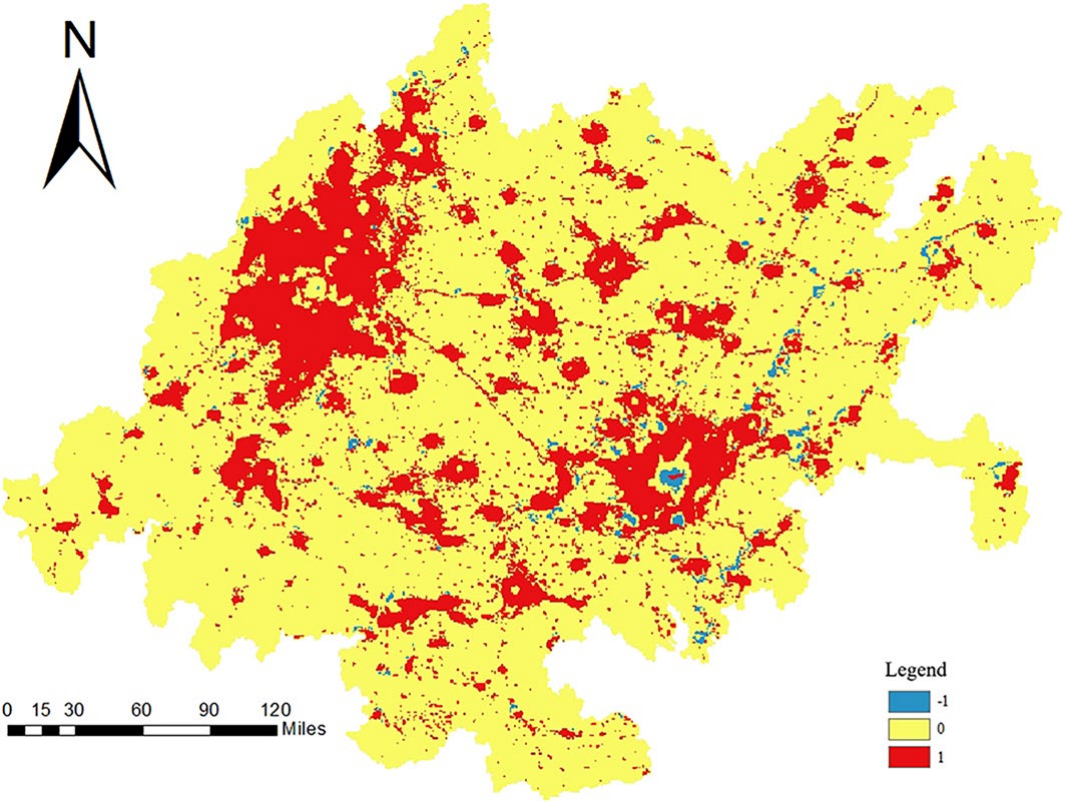
Trend of carbon emissions.

**Figure 13:**
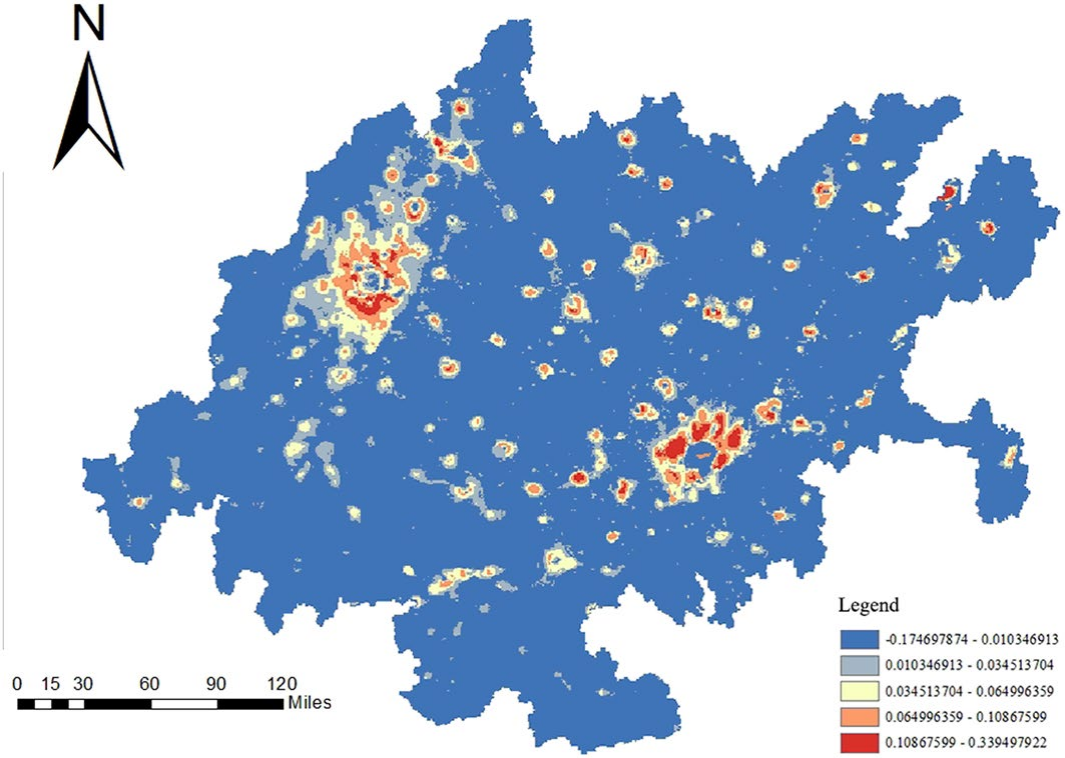
Magnitude of carbon emission changes.

Figure 12 shows the visualization results of the trend analysis. The carbon emissions in the CCEZ from 2000 to 2020 experienced an overall upward trend with stable changes. From the perspective of the internal regions, the areas with significant carbon emission changes are mainly concentrated in the northwestern and southeastern central regions. The trend in carbon emissions in the northwest region is generally greater than 0, indicating an upward trend in carbon emissions, while the trend in carbon emissions in the southeast central region is less than 0, indicating a significant downward trend. The Mann‒Kendall trend test also revealed the magnitude of carbon emission changes in the CCEZ, revealing that the overall change from 2000 to 2020 was relatively small and the results are shown in Fig. 13.

## Discussion and conclusions

This paper presents a carbon emission estimation model for the CCEZ in China constructed based on combined, corrected NPP-VIIRS and DMSP/OLS nighttime light data. Utilizing a combined qualitative and quantitative research framework, we analyze the spatiotemporal evolution and disparities of carbon emissions at multiple scales (provincial, municipal, county, and grid) from 2000 to 2020. Qualitative analysis encompasses assessments of total carbon emission growth, average annual growth rates, and changes in high- and low-carbon emission regions. Quantitative analysis includes spatial autocorrelation analysis and Mann‒Kendall trend analysis, among others. The main findings are as follows:

In terms of carbon emission fitting, from 2000 to 2020, the estimated model between the corrected total nighttime light value and the county-scale carbon emissions in the CCEZ has an R^2^ greater than 0.8, indicating a significant positive correlation between the two. The root mean square error (RMSE) and mean relative error (MRE) between the estimated and statistical values of carbon emissions in the CCEZ are small, indicating the credibility of the fitted equation in estimating carbon emissions from 2000 to 2020. Therefore, nighttime light data can be used to study the spatialization of carbon emissions in the CCEZ.

The qualitative analysis of the spatiotemporal evolution pattern of carbon emissions in the CCEZ revealed that (i) at the provincial and municipal scales, carbon emissions in the CCEZ continued to increase from 2000 to 2020, an increase of 2.5 times. The carbon emissions in Chengdu and Chongqing are significantly greater than those in other cities. The number of areas with lower carbon emissions and higher carbon emissions within the CCEZ has increased, with a trend of expansion toward the north for areas with higher carbon emissions and a trend of expansion toward the south for areas with lower carbon emissions. (ii) At the county scale, in terms of total carbon emissions, counties with high carbon emissions account for 1–6%. The areas with lower carbon emissions are concentrated in the edge cities of the Chengdu-Chongqing urban agglomeration and cities with lower industrialization levels around the Sichuan Basin, with low carbon emission counties accounting for 21–41%. Over time, the high carbon emission areas in the northern region decreased, forming a spatial structure of two core areas with higher carbon emissions in the east and west and surrounding areas with lower carbon emissions. (iii) At the grid scale, from 2000 to 2020, the high carbon emission areas in the CCEZ exhibited a multicore distribution pattern, evolving into two cores with Chengdu and Chongqing as the centers, gradually expanding radially outward, and ultimately forming a connected pattern of high carbon emission core areas.

Quantitative analysis of the spatiotemporal evolution pattern of carbon emissions in the CCEZ revealed the following: (i) In terms of spatial autocorrelation analysis, county-scale and raster-scale carbon emissions in the CCEZ from 2000 to 2020 exhibited significant global spatial positive spatial correlations. (ii) In terms of hotspot analysis, the distribution of carbon emission hotspots shows a significant dual-core outward diffusion structural evolution trend. At the county level, extreme hotspot areas are in a state of aggregation. The hotspots and subhotspots are adjacent to the extreme hotspots, indicating a radiation effect from high carbon emission county-scale units to their surroundings. The coldspots and subcoldspots are distributed in the edge cities of the CCEZ with lower industrialization levels around the Sichuan Basin. At the grid scale, in 2000, the hotspot distribution showed a scattered structure centered on the main urban areas of Chengdu and Chongqing. In 2013, the structure of the hotspot areas changed, forming an axis band radiating outward with the main urban areas of Chengdu and Chongqing as the core high carbon emission areas. In 2020, the carbon emission hotspot areas continued to expand around the core areas. (iii) According to the Lisa cluster analysis, at the county scale, the CCEZ has experienced an agglomeration trend caused by high-carbon agglomeration areas and low-carbon agglomeration areas. The high-carbon agglomeration areas are relatively stable in more than 30 counties in Chengdu, Deyang, and Chongqing city, while the low-carbon agglomeration areas are located in the southwest and central parts of the CCEZ. (iv) According to the Mann‒Kendall trend analysis, the carbon emissions in the CCEZ from 2000 to 2020 exhibited an overall upward trend and a stable change, which was related to factors such as regional energy consumption and industrial structure.

Based on the above research, we should consider the distribution and agglomeration of carbon emissions in different economic centers and at different scales and formulate carbon emission reduction strategies according to local conditions. Different economic centers, such as the CCEZ, the Pearl River Delta Economic Circle and the Beijing–Tianjin–Hebei Economic Circle (BTHC), are important regions for China’s economic development, but there are still some differences in their carbon emission trends. In terms of total carbon emissions, compared with those in other economic zones, the total carbon emissions in the CCEZ are relatively low, which may be attributed to its relatively singular industrial structure and relatively low population density. The total carbon emissions of the BTHC are relatively large, which may be affected by the dominant position of heavy industry and service industry and the high population density. In terms of the growth rate of carbon emissions, compared with that in other economic zones, the growth rate of carbon emissions in the CCEZ is relatively slow, which may be affected by environmental protection policies and promoted by industrial restructuring. In the past two years, the growth rate of carbon emissions has accelerated, which may be affected by rapid economic growth. In terms of carbon emission agglomeration characteristics, the carbon emissions within the CCEZ show relatively scattered agglomeration characteristics. The high-carbon emission areas are mainly concentrated in major cities and their surrounding areas, while the low-carbon emission areas are mainly distributed in the edge areas of urban agglomerations. The main reason is that the industrial structure of the region is relatively diversified but relatively scattered, including manufacturing, the service industry, agriculture and other fields. Although industrial activities in major cities and their surrounding areas lead to higher carbon emissions, carbon emissions are relatively dispersed due to the diversity of the overall industrial layout. The concentration of carbon emissions within the BTHC is relatively high, possibly because the urbanization process accelerates population agglomeration and industrial agglomeration, which in turn leads to more concentrated carbon emissions.

Therefore, we need to formulate differentiated carbon emission reduction strategies according to the characteristics of the carbon emission distribution in different economic centers. For example, for the high-carbon agglomeration area within the CCEZ, we should accurately locate the emission reduction area, adjust the industrial structure, and increase scientific and technological innovation and governance. For the low-carbon agglomeration area within the CCEZ, it is necessary to consolidate and develop its own advantages and optimize the industrial structure and energy structure. At the same time, we should strengthen the multiscale management of the CCEZ and take corresponding management measures according to the distribution of carbon emissions at different scales (provincial, municipal, county and grid levels). For example, at the county level, we can focus on carbon emissions; at the grid scale, refined carbon emission monitoring and governance can be implemented. In addition, according to the Mann‒Kendall trend test results, carbon emission targets are set to limit the growth of carbon emissions to mitigate climate change.

However, there are several limitations in this study, such as the relative simplification of the model and the failure to consider changes in the industrial structure. Future research can expand the time range, introduce more factors to construct a multifactor model, evaluate the effectiveness of carbon emission reduction policies, and conduct cross-regional collaborative research.

## Data Availability

The data that support the findings of this study are available from the corresponding author upon reasonable request.
